# The correlation between intrathoracic herniation of the gastric tube and postoperative complications and the efficacy of laparoscopic retrosternal route creation

**DOI:** 10.1007/s10388-025-01119-6

**Published:** 2025-03-20

**Authors:** Yutaka Kishimoto, Koji Otsuka, Takeshi Yamashita, Akira Saito, Masahiro Kohmoto, Kentaro Motegi, Tomotake Ariyoshi, Satoru Goto, Masahiko Murakami, Takeshi Aoki

**Affiliations:** 1 Department of Gastrointestinal and General Surgery, School of Medicine, Showa University, Tokyo, Japan; 2 Esophageal Cancer Center, Showa University Hospital, 1-5-8, Hatanodai, Sinagawa-ku, Tokyo 142-8666 Japan

**Keywords:** Delayed gastric conduit emptying, Esophagectomy, Intrathoracic herniation, Postoperative complication, Retrosternal route

## Abstract

**Background:**

Gastric tube prolapse into the thoracic cavity in retrosternal route reconstruction during esophagectomy is known as intrathoracic herniation of the gastric tube (IHGT). However, few reports have determined whether a correlation exists between IHGT and postoperative complications. Moreover, the optimal procedure for avoiding IHGT remains unclear.

**Methods:**

This retrospective study included 200 patients who had undergone subtotal esophagectomy and retrosternal gastric tube reconstruction at a single institution. The diagnosis of IHGT was defined as a gastric tube herniation length of ≥ 5 cm on plain chest radiography. The frequency of postoperative complications in patients with IHGT was measured to determine any correlation. The incidence of IHGT in a laparoscopic retrosternal route creation group was also measured and the efficacy of this procedure was investigated.

**Results:**

The overall incidence of IHGT was 7.5%. The incidence of grade II or higher anastomotic leakage and atelectasis was significantly higher in the IHGT( +) (anastomotic leakage, 26.7% vs. 4.3%, *P* = 0.007; atelectasis, 40.0% vs. 13.5%, *P* = 0.016). In univariate analysis, IHGT( +) showed a significantly higher incidence of anastomotic leakage (OR 7.88, *P* = 0.007). In multivariate analysis, IHGT was an independent risk factor for atelectasis (OR 5.03, *P* = 0.005). Furthermore, the incidence of IHGT was significantly lower in the laparoscopic group (2.0% vs. 13.0%, *P* = 0.005).

**Conclusion:**

Our findings show that IHGT may be correlated with grade II or higher anastomotic leakage and atelectasis. Laparoscopic retrosternal route creation may be effective in avoiding IHGT and contributes to a reduction in postoperative complications.

## Introduction

The retrosternal route is widely used for reconstruction route after esophagectomy. Perforation of the mediastinal pleura owing to blind dissection during the creation of the retrosternal route and the resulting prolapse and dilation of the gastric tube into the thoracic cavity has been termed intrathoracic herniation of the gastric tube (IHGT), with concerns raised about its association with postoperative complications. IHGT is a complication occasionally observed after esophagectomy; however, its clinical effect is unclear.

A correlation between IHGT and postoperative complications has been reported [1]; however, most reports have been case studies. Furthermore, the methods to avoid IHGT are also not clear.

Conventionally, blunt dissection with intestinal spatula has been used for creating a retrosternal route. However, based on the experience of patients who developed anastomotic leakage (AL) from postoperative IHGT, we recently standardized a technique for laparoscopic retrosternal route creation. In this study, we aimed to investigate the correlation between IHGT and postoperative complications and determine whether the laparoscopic retrosternal route was effective in avoiding IHGT.

## Methods

### Patients and methods

This retrospective study included 200 patients who had undergone subtotal esophagectomy and retrosternal gastric tube reconstruction for the preoperative diagnosis of esophageal cancer at Showa University Hospital Esophageal Cancer Center (Tokyo, Japan) between August 2020 and September 2023. The frequency of postoperative complications in patients with IHGT that had occurred within the above period was measured and multivariate analysis was used to determine whether there was a correlation between IHGT and the occurrence of postoperative complications. Furthermore, the incidence of IHGT was compared among the different procedures used to create the retrosternal route. We used the Clavien–Dindo classification [2] grade II or higher to measure postoperative complications. Data concerning albumin levels and body mass index (BMI) measured 4 days prior to surgery were collated. Comorbidities were assessed according to the following definitions:

1. Cardiac dysfunction: ejection fraction < 50% on preoperative ultrasound cardiography was considered significant.
2. Pulmonary dysfunction: forced expiratory volume1.0% < 70% on preoperative pulmonary function tests was considered significant.
3. Renal dysfunction: creatinine clearance (CCr) < 60 was considered significant. CCr was calculated using the Cockcroft–Gault equation, with creatinine values included from the measurements 4 days before surgery.
4. Diabetes mellitus: HbA1c > 6.5% from the data 4 days before surgery was considered significant.

The study protocol was approved by the Ethics Committee of Showa University (Accession No. 2023–201-A). All patients provided written informed consent for their clinical information to be used for future medical research prior to surgery.

### Surgical procedures

The thoracic procedure was performed using video- or robot-assisted thoracoscopic surgery in all cases [3]. The abdominal procedure was performed using a hand-assisted laparoscopic surgery technique with three ports and a 7-cm transverse incision in the upper abdomen. For the gastric tube, the right gastroepiploic artery was preserved along its entire length, while the left gastroepiploic artery was divided at its terminal branch, exposing the gastric wall. From this point orally, the great omentum was dissected along the gastric wall. A subtotal gastric tube with a diameter of 5 cm was created using a linear stapler. Anastomosis was performed in the neck in all cases, with circular stapler anastomosis or hand-sewn method as the standard technique. After completing the anastomosis, the gastric tube was pulled down into the abdominal cavity to straighten it and eliminate any slack [4]. We do not place gastrostomy feeding tubes in routine practice, and postoperative nasogastric tubes were not placed in any case.

To create the retrosternal route, a peritoneal incision was first made four finger widths caudal to the xiphoid process from the small laparotomy wound. Sharp dissection was performed along the sternum under direct visualization to reach the retrosternal space. In the conventional method, while taking care to avoid pleural injury, a 20-mm intestinal spatula was used to bluntly dissect the retrosternal space without exceeding the width of the sternum and this space was extended up to the neck. In the laparoscopic creation method, under a pneumoperitoneum, the retrosternal space was bluntly or sometimes sharply dissected using laparoscopic coagulation shears while visualizing the procedure on a monitor. If possible, the procedure was completed after confirming penetration of the tunnel up to the neck.

### Diagnosis of IHGT

**Fig. 1:**
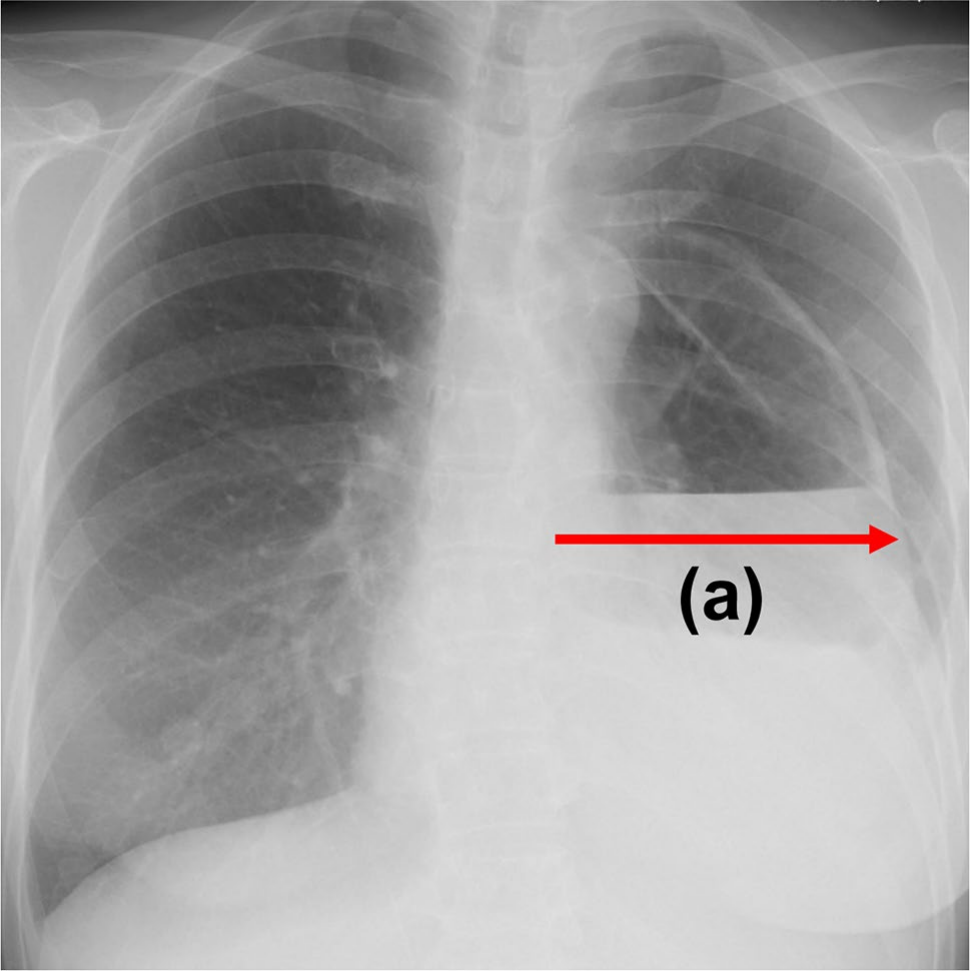
Cases in which the distance from the lateral edge of vertebra to the edge of the dilated gastric tube (**a**) was ≥ 5 cm on plain chest radiographs were defined as intrathoracic herniation of the gastric tube-positive

The diagnosis of IHGT was defined, based on a report by Uemura et al. [1], as a gastric tube herniation length (distance from the lateral edge of the vertebral body to the edge of the herniated gastric tube) of ≥ 5 cm on frontal plain chest radiography **(**Fig. 1**)**. Patients who met the above criteria within 7 days of surgery were diagnosed as IHGT( +).

### Statistical analysis

A Mann–Whitney *U* test was used for the analysis of continuous variables and Fisher’s exact test was used for the analysis of categorical variables. To identify the factors associated with postoperative complications, multivariate analyses using a stepwise logistic regression were performed. We determined the number of variables to be used in the multivariate analysis by adhering to the rule that requires a minimum of 10 events per variable for each predictor variable in logistic regression models [5]. When multivariate analysis could not be performed because of an insufficient number of events, only univariate analysis was conducted. All statistical analyses were performed with EZR (Saitama Medical Center, Jichi Medical University, Saitama, Japan) software [6], and a *P-*value < 0.05 was considered statistically significant.

## Results

### Patient characteristics

In total, 200 patients had undergone subtotal esophagectomy and retrosternal gastric tube reconstruction. Postoperative IHGT was observed in 15 (7.5%) patients. The clinical characteristics of the patients are summarized in Table 1. The patient characteristics did not differ significantly between the two groups.

**Table 1:** Characteristics of 200 patients according to IHGT

Variables	IHGT(-) (*n* = 185)	IHGT( +) (*n* = 15)	*P* value
Age(years);median (range)	69 (45–87)	66 (44–82)	0.58
Gender,male /female	147 (79.5%)/38 (20.5%)	12 (80.0%)/3 (20.0%)	1.00
Preoperative laboratory data			
BMI, median (range)	21.2 (14.4–33.7)	22.4 (14.0–27.9)	0.28
Alb, g/dl median (range)	3.9 (2.6–4.7)	3.9 (3.3–4.6)	0.58
Tumor location^a^			0.42
Ce	14 (7.6%)	0 (0%)	
Ut	19 (10.3%)	0 (0%)	
Mt	81 (43.8%)	7 (46.7%)	
Lt	49 (26.5%)	6 (40.0%)	
Jz	22 (11.9%)	2 (13.3%)	
Preoperative therapy			0.32
NAC	134 (72.5%)	11 (73.4%)	
None	24 (13.0%)	0 (0%)	
ESD	12 (6.5%)	2 (13.3%)	
CRT	15 (8.0%)	2 (13.3%)	
Comorbidity			
Cardiac function EF (< 50% / EF > 50%)	3 (1.6%)/182 (98.4%)	0 (0%)/15 (100.0%)	1.00
Respiratory function FEV1.0% (< 70% / > 70%)	70 (37.8%)/115 (62.2%)	3 (20.0%)/12 (80.0%)	0.26
Renal function CCr (< 60 ml/min / > 60 ml/min)	39 (21.1%)/146 (78.9%)	2 (13.3%)/13 (86.7%)	0.74
Diabetes mellitus HbA1c (> 6.5%) ( +)/(−)	38 (20.5%)/147 (79.5%)	3 (20.0%)/12 (80.0%)	1.00

*IHGT* intrathoracic herniation of gastric tube, *BMI* body mass index, *Alb* albumin, *Ce* cervical esophagus,

*NAC* neoadjuvant chemotherapy, *ESD* endoscopic submucosal dissection, *CRT* chemoradiotherapy,

*EF* ejection fraction, *CCr* creatinine clearance

^a^Japanese Classification of Esophageal Cancer (12th edition)

The surgical outcomes of patients are summarized in Table 2. The incidence of IHGT was significantly lower in the laparoscopic retrosternal route creation group than in the conventional group (2.0% vs. 13.0%, respectively; *P* = 0.005).

**Table 2:** Surgical outcomes of 200 patients according to IHGT

Variables	IHGT(−) (*n* = 185)	IHGT( +) (*n* = 15)	*P* value
Surgical approach of thoracic part			0.31
VATS	146 (78.9%)	14 (93.3%)	
Robot assisted	39 (21.1%)	1 (6.7%)	
Retrosternal route creation			0.005
Conventional	87 (47.0%)	13 (86.7%)	
Laparoscopic	98 (53.0%)	2 (13.3%)	
Anastomosis			1.00
Circular stapler	182 (98.4%)	15 (100.0%)	
Hand-sewn	3 (1.6%)	0 (0%)	
Number of fields of lymphadenectomy^a^			1.00
2	96 (51.9%)	8 (53.3%)	
3	89 (48.1%)	7 (46.7%)	
Operation time,min Median (range)	440 (179–834)	398 (201–646)	0.36
Blood loss,ml Median (range)	141 (5–1092)	140 (26–1130)	0.99

*IHGT* intrathoracic herniation of gastric tube*, VATS* video-assisted thoracoscopic surgery

^a^Japanese Classification of Esophageal Cancer (12th edition)

### *Details of IHGT(* +*) patients*

The clinical course and surgical details of the IHGT( +) patients are summarized in Table 3.

**Table 3:** Characteristics of the patients with IHGT( +)

Patient	Gender	Age	Date of diagnosis of IHGT	Symptom	Date of diagnosis of AL	Date of diagnosis of Atelectasis	NG tube insertion	Start of oral feeding	Reoperation
1	Male	44	1POD	–	–	–	–	7POD	–
2	Male	66	2POD	dyspnea	–	3POD	–	7POD	–
3	Male	75	5POD	dyspnea	12POD	7POD	–	12POD	–
4	Male	47	2POD	–	–	–	–	10POD	–
5	Male	67	2POD	dyspnea	–	3POD	–	9POD	–
6	Male	74	2POD	nausea	–	–	+	11POD	–
7	Male	50	4POD	–	7POD	–	–	13POD	–
8	Male	58	4POD	–	–	–	–	7POD	–
9	Male	61	3POD	–	11POD	–	–	19POD	–
10	Male	58	2POD	dyspnea	–	3POD	–	8POD	–
11	Male	51	2POD	–	–	–	–	5POD	–
12	Male	73	1POD	dyspnea	13POD	2POD	–	9POD	–
13	Female	75	3POD	dyspnea	–	4POD	–	17POD	–
14	Female	58	1POD	-	-	-	-	5POD	-
15	Female	82	4POD	-	-	-	-	7POD	-

*IHGT* intrathoracic herniation of gastric tube, *POD* postoperative days, *AL* anastomotic leakage, *NG tube* nasogastric tube

In this study, no patient required reoperation, but one (6.7%) patient needed nasogastric tube insertion postoperatively. An 8-Fr decompression tube was inserted on postoperative day 3 and remained in place until postoperative day 10.

### The correlation between IHGT and postoperative complications

Postoperative complications are shown in Table 4. AL was significantly more frequent in the IHGT( +) group [26.7% vs. 4.3%, respectively; *P* = 0.007]. Atelectasis was also significantly more frequent in the IHGT( +) group [40.0% vs. 13.5%, respectively; *P* = 0.016].

**Table 4:** Postoperative complications^a^ according to IHGT

Variables	IHGT(−)	IHGT( +)	*P* value
	(*n* = 185)	(*n* = 15)	
Anastomotic leakage	8 (4.3%)	4 (26.7%)	0.007
Atelectasis	25 (13.5%)	6 (40.0%)	0.016
Pneumonia	9 (4.9%)	1 (6.7%)	0.55
Arrhythmia	21 (11.4%)	4 (26.7%)	0.17

^a^Clavien–Dindo classification Grade 2 or higher, *IHGT* intrathoracic herniation of gastric tube

### Univariate analysis of factors associated with AL

AL was observed in 12 (6.0%) patients; thus, multivariate analysis could not be performed [5].

In univariate analysis, IHGT( +) was identified as the only significant risk factor for AL ([OR] 7.88, *P* = 0.007) **(**Table 5**)**.

**Table 5:** Univariate analysis of factors associated with anastomotic leakage

Variables	Univariable analysis		
	OR	95% CI	*P* value
Age (> median)	1.83	0.47–8.60	0.38
Gender (male)	1.31	0.26–12.75	1
BMI (> median)	2.12	0.55–9.97	0.25
Alb (< median)	0.83	0.20–3.16	1
Tumor Location^a^ Ce (vs others)	1.22	0.03–9.73	0.59
Anastomosis (Circular stapler)	0	0.00–40.05	1
Operation time (> median)	0.69	0.17–2.61	0.57
Blood loss (> median)	1.46	0.38–6.04	0.57
IHGT ( +)	7.88	1.50–35.63	0.007
Preoperative therapy CRT (vs others)	2.29	0.22–12.33	0.27
Comorbidity			
Cardiac dysfunction EF < 50%	0	0.00–40.05	1
Respiratory dysfunction FEV1.0% < 70%	0.33	0.03–1.62	0.22
Renal dysfunction CCr < 60 ml/min	1.32	0.22–5.60	0.71
Diabetes mellitus HbA1c > 6.5%	0.77	0.08–3.81	1

*BMI* body mass index*, Alb* albumin, *Ce* cervical esophagus, *IHGT* intrathoracic herniation of gastric tube, *CRT* chemoradiotherapy, *EF* ejection fraction, *CCr* creatinine clearance

^a^Japanese Classification of Esophageal Cancer (12th edition)

### Multivariate analysis of factors associated with atelectasis

Atelectasis was observed in 31 (15.5%) patients and multivariate analysis was performed [5].

Multivariate analysis showed that tumor location in the cervical esophagus (Ce) and IHGT( +) were significantly associated with grade II or higher atelectasis [Ce: OR 4.19, *P* = 0.018; IHGT( +): OR 5.03, *P* = 0.005; Table 6].

**Table 6:** Univariate and multivariate analysis of factors associated with atelectasis

Variables	Univariable analysis			Multivariable analysis		
	OR	95% CI	*P* value	OR	95% CI	*P* value
Age (> median)	1.09	0.47–2.56	0.85			
Gender (male)	2.7	0.77–14.61	0.15			
BMI (> median)	0.93	0.40–2.15	1			
Alb (< median)	0.7	0.29–1.64	0.44			
Tumor Location^a^ Ce (vs others)	3.39	0.83–12.37	0.046	4.19	1.28–13.80	0.018
Anastomosis (Circular stapler)	2.76	0.04–54.6	0.39			
Operation time (> median)	1.23	0.53–2.88	0.69			
Blood loss (> median)	1.5	0.65–3.56	0.33			
IHGT ( +)	4.22	1.13–14.67	0.016	5.03	1.62–15.60	0.005
Preoperative therapy CRT (vs others)	1.19	0.21–4.65	0.73			
Comorbidity						
Cardiac dysfunction EF < 50%	0	0.00–13.40	1			
Respiratory dysfunction FEV1.0% < 70%	0.95	0.38–2.24	1			
Renal dysfunction CCr < 60 ml/min	1.16	0.39–3.07	0.81			
Diabetes mellitus HbA1c > 6.5%	1.16	0.39–3.07	0.81			

*BMI* body mass index, *Alb* albumin, *Ce* cervical esophagus, *IHGT* intrathoracic herniation of gastric tube, *CRT* chemoradiotherapy, *EF* ejection fraction, *CCr* creatinine clearance

^a^Japanese Classification of Esophageal Cancer (12th edition)

### Univariate analysis of factors associated with IHGT

IHGT was observed in 15 (7.5%) patients; thus, multivariate analysis could not be performed [5].

In univariate analysis, conventional retrosternal route creation was associated with a significantly higher incidence of IHGT ([OR] 7.26, *P* = 0.005) **(**Table 7**)**.

**Table 7:** Univariate analysis of factors associated with IHGT

Variables	Univariable analysis		
	OR	95% CI	*P* value
Age (> median)	0.42	0.11–1.40	0.18
Gender (male)	1.03	0.26–5.99	1
BMI (> median)	1.55	0.47–5.50	0.59
Comorbidity			
Cardiac dysfunction EF < 50%	0	0.00–31.1	1
Respiratory dysfunction FEV1.0% < 70%	0.41	0.07–1.60	0.26
Renal dysfunction CCr < 60 ml/min	0.58	0.06–2.71	0.74
Diabetes mellitus HbA1c > 6.5%	0.96	0.17–3.84	1
Retrosternal route creation (Conventional)	7.26	1.58–68.2	0.005
Operation time (> median)	0.47	0.11–1.56	0.19
Blood loss (> median)	0.89	0.26–2.92	1

*IHGT* intrathoracic herniation of gastric tube*, BMI* body mass index, *EF* ejection fraction, *CCr* creatinine clearance

## Discussion

Herniation of a reconstructed organ into the thoracic cavity after retrosternal reconstruction during esophagectomy is a relatively common postoperative complication [7–14]. Even if herniation of the reconstructed organ occurs, it rarely causes symptoms of passage obstruction or becomes a clinical problem. However, a small number of cases require reoperation when conservative treatment is ineffective. In reports of cases requiring reoperation owing to prolonged passage obstruction, the surgical procedures have varied widely, with none of them being unchallenging [8–14]. While the frequency of IHGT is not high, it is important to avoid IHGT, considering the complexity of the surgical procedure when reoperation is required.

The exact mechanism of IHGT is not clearly understood, however, many reports suggest that it may be caused by perforation of the parietal pleura during the creation of the retrosternal route [8–13]. This results in the gastric tube being drawn into the thoracic cavity through negative pressure and expanding into a sac-like shape. In our study, the incidence of IHGT was significantly lower in the laparoscopic group, and this procedure may be effective in preventing IHGT. Horikawa et al. [15] also reported the usefulness of laparoscopic retrosternal route creation. This procedure is advantageous in that dissection of the retrosternal route can be performed under direct visualization, which reduces the risk of pleural injury. Additionally, even if pleural injury occurs, tearing and opening of the site are avoided, which can reduce the risk of gastric tube herniation. Moreover, bleeding from blunt dissection of the retrosternal space can be controlled via direct visualization. In this study, pleural injury was confirmed during the procedure in both the IHGT cases in the laparoscopic group. These incidents occurred in the early stages of laparoscopic procedure introduction, and they likely occurred due to a lack of technical proficiency at that time. However, in more recent cases, the frequency of pleural injury during the procedure has decreased, suggesting that the occurrence of IHGT is being prevented.

Correlation factors for AL after esophageal cancer surgery have been reported to include BMI, tumor location, albumin level, and comorbidities such as diabetes mellitus and COPD [16, 17]. Furthermore, correlation factors for pulmonary complications have been reported to include BMI, age, intraoperative blood loss, and comorbidities such as diabetes mellitus, cardiac dysfunction, COPD, and operation time [18, 19]. Our study suggests a potential correlation between IHGT and the incidence of AL and atelectasis. As shown in Table 3, IHGT occurred prior to AL and atelectasis. Therefore, we believe that IHGT is a triggering factor for these postoperative complications.

In our study, the frequency of AL was significantly higher in the IHGT( +) group. Sutcliffe et al. [20] reported similar results and speculated that gastric tube dilation causes twisting or tension at the anastomosis, inducing ischemia and leading to impaired healing of the anastomosis, thereby promoting AL. We have also encountered cases in which gastric tube dilation caused excessive tension on the anastomosis and impaired blood flow, which likely worsened a minor anastomotic leak into becoming a major leak.

In our study, a significant correlation was observed between IHGT and atelectasis. Benedix et al. [21] reported that delayed gastric emptying (DGE) after esophagectomy correlated with respiratory complications, but the mechanism involved has not been investigated. We consider that atelectasis is caused by physical compression of the lungs due to the expanded gastric tube. Although cervical esophageal cancer has also been identified as an independent risk factor for atelectasis, we could not find any study specifically mentioning this. It is thought that a higher tumor location is associated with more inflammatory changes around the trachea caused by surgical procedure, potentially resulting in increased airway secretions and subsequent atelectasis.

This study has some limitations. First, this was a single-center retrospective study and due to the insufficient number of events, we were unable to perform multivariate analysis of the risk factors for anastomotic leakage and IHGT. Second, while the definition of IHGT in this study corresponds to early delayed gastric conduit emptying (DGCE) in the international expert consensus on DGCE after esophagectomy [22], the diagnosis is not strictly the same. The international consensus defines early DGCE as an increase in the width of the gastric tube on plain radiography of ≥ 100% compared with the width of the gastric tube on the day of surgery, in addition to the presence of an air-fluid level. However, identifying the width of the gastric tube on radiography on the day of surgery is difficult. Therefore, based on Uemura et al.’s report [1], we defined IHGT-positive as a gastric tube herniation length of ≥ 5 cm and evaluated it accordingly. Third, this study was initiated based on our institution's experience that early incidence of IHGT were associated with more postoperative complications, therefore, cases of late incidence of IHGT were not examined. As there are concerns that late incidence of IHGT may also affect the postoperative course, further investigation is necessary in future.

## Conclusion

IHGT after esophagectomy may be a risk factor for AL and atelectasis. Laparoscopic retrosternal route creation may be effective in avoiding IHGT and can be expected to reduce postoperative complications.
